# Extensive data engineering to the rescue: building a multi-species katydid detector from unbalanced, atypical training datasets

**DOI:** 10.1098/rstb.2023.0444

**Published:** 2024-06-24

**Authors:** Shyam Madhusudhana, Holger Klinck, Laurel B. Symes

**Affiliations:** ^1^ Centre for Marine Science and Technology, Curtin University, Perth, Western Australia 6845, Australia; ^2^ K. Lisa Yang Center for Conservation Bioacoustics, Cornell Lab of Ornithology, Cornell University, Ithaca, NY 14853-0001, USA; ^3^ Smithsonian Tropical Research Institute, Balboa, Ancón, Panama City 0843-03092, Republic of Panama

**Keywords:** passive acoustic monitoring, machine learning, katydid, data engineering, bioacoustics, tropics

## Abstract

Passive acoustic monitoring (PAM) is a powerful tool for studying ecosystems. However, its effective application in tropical environments, particularly for insects, poses distinct challenges. Neotropical katydids produce complex species-specific calls, spanning mere milliseconds to seconds and spread across broad audible and ultrasonic frequencies. However, subtle differences in inter-pulse intervals or central frequencies are often the only discriminatory traits. These extremities, coupled with low source levels and susceptibility to masking by ambient noise, challenge species identification in PAM recordings. This study aimed to develop a deep learning-based solution to automate the recognition of 31 katydid species of interest in a biodiverse Panamanian forest with over 80 katydid species. Besides the innate challenges, our efforts were also encumbered by a limited and imbalanced initial training dataset comprising domain-mismatched recordings. To overcome these, we applied rigorous data engineering, improving input variance through controlled playback re-recordings and by employing physics-based data augmentation techniques, and tuning signal-processing, model and training parameters to produce a custom well-fit solution. Methods developed here are incorporated into Koogu, an open-source Python-based toolbox for developing deep learning-based bioacoustic analysis solutions. The parametric implementations offer a valuable resource, enhancing the capabilities of PAM for studying insects in tropical ecosystems.

This article is part of the theme issue ‘Towards a toolkit for global insect biodiversity monitoring’.

## 1. Introduction

Recent advances in data processing have ushered in transformative changes across various biological disciplines. Among these, the realm of acoustic signal detection and classification in soundscape recordings offers immense potential, with the capacity to yield unparalleled insights into spatio-temporal occurrence patterns of species within ecosystems (e.g. [1]). Over the past few decades, passive acoustic monitoring (PAM) methods using automatic signal recognition have been widely employed for monitoring a variety of terrestrial and aquatic fauna [2,3]. In the past decade, the adoption of machine learning (ML)-based automation approaches, which typically offer superior accuracy and robustness [4] over conventional techniques, has greatly enhanced the success of PAM methods in monitoring fauna at scale (e.g. [5]). Despite the demonstrated successes, there have been fewer studies employing ML-driven PAM approaches for monitoring insect populations.

Comprehensive insights into the composition of insect communities offer invaluable data for the purposes of conservation and effective management [6,7]. Unsettled debates regarding the nature and extent of declines in insect populations underscore our limited knowledge concerning this class of fauna, which occupy a key trophic level in the food chain [8,9]. The scarcity of knowledge includes critical details such as seasonality and distribution patterns across the forest canopy and understory [10,11]. In evolving forest ecosystems and amidst diminishing insect populations, persistent assessment of insect populations will be key for generating interpretable and actionable data. In agricultural applications such as detecting crop pests and adaptively modulating deployment of pesticides and other control measures, adoption of automated PAM techniques can facilitate faster interventions.

**Figure 1.**
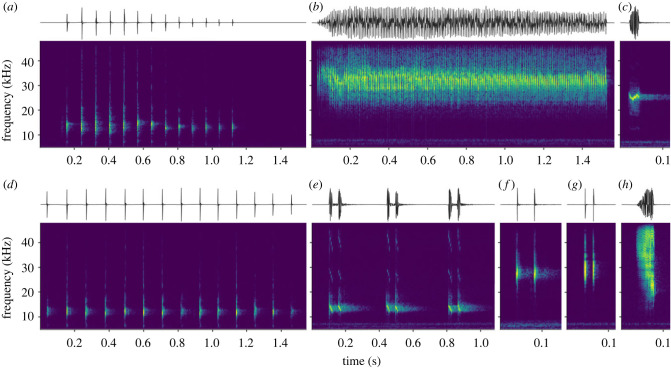
Waveforms and spectrograms demonstrating inter-specific distinctiveness in calls of a few of the many species of katydids found at the study site: (*a*) *Euceraia atryx*, (*b*) *Erioloides longinoi*, (*c*) *Docidocercus gigliotosi*, (*d*) *Euceraia insignis*, (*e*) *Ischnomela pulchripennis*, (*f*) *Anaulacomera spatulata*, (*g*) *Anaulacomera furcata* and (*h*) *Montezumina bradleyi*.

Insect calls offer a proxy for their spatial and temporal dynamics [12]. However, insects pose unique challenges for acoustic monitoring. Chorusing insects, such as several species of crickets, are generally more straightforward to detect due to their repetitive and synchronized calling patterns [13]. By contrast, katydids are not typically associated with the formation of choruses. Some employ a strategy that seeks to strike a balance between communication with conspecifics and minimizing their exposure to potential predators [14–16], often leading to low signalling rates. In addition, katydid calls and their calling behaviour are the result of a complex interplay of various selective forces. These include the preferences of females [17–19], competition among males [20,21], trade-offs between female preferences and male–male competition [22,23], the influence of parasites [24–26], the effects of environmental features on signal transmission [27–30] and energy constraints [31]. The resulting temporal, spectral and spectro-temporal niche-partitioning (e.g. [32]; figure 1) drive a greater degree of distinctiveness and dynamism in their acoustic behaviour, rendering the automated recognition of katydid species a more challenging task.

In this study, we undertook the development of a convolutional neural network (CNN)-based solution to automate the recognition of 31 katydid species of interest found on the Barro Colorado Island (BCI), situated within the Panama Canal. BCI is home to a diverse community of over 80 katydid species, each contributing to the soundscape with calls spanning a wide bandwidth, including high ultrasonics, and varying in duration from mere milliseconds to multiple seconds [33]. Notably, certain katydid species emit calls with intricate multi-pulse structures, where subtle differences in inter-pulse intervals or central frequencies serve as the only discernible features. These nuanced differences, coupled with lower source levels and higher susceptibility to masking (even under moderate ambient noise), make species identification in PAM recordings a very challenging endeavour. Further complicating matters, our initial training dataset was limited and characterized by significant class imbalances. To overcome these challenges, we engaged in extensive data engineering, implementing a range of techniques to expand class representation and variance in the training dataset. We carefully selected approaches and parameters for data preprocessing, class balancing, input conditioning and data augmentations. Each of these choices was informed by both practical considerations and domain awareness on katydid call diversity.

Another serious challenge to realising an ML solution was the atypical nature of the available training recordings—clean focal recordings that were gathered by placing captured individuals in controlled settings presented a stark domain mismatch with real-world soundscapes where the model would ultimately be deployed. Though often not as pronounced, domain mismatch problems are commonly encountered in bioacoustic ML, across various taxa including insects (e.g. [34,35]), birds (e.g. [36,37]) and marine fauna (e.g. [38,39]). Consequently, a variety of solutions have also been explored, including the use of transfer learning following a pretraining using general-purpose datasets [37,40,41], source separation and denoising [42,43], discriminative training [44], domain adaptation based on covariance normalization [45], unsupervised domain adaptation [46,47], domain/context adaptive neural networks [36,48], etc. Use of data augmentation techniques to address domain mismatch problems was common among the top-performing solutions in a public challenge focused on developing generalizable methods for detecting birds [49]. Besides expanding on the available recordings, our solution also relied largely on using augmentations. We considered physics-based augmentations, in both time- and spectral domains, to produce near-realistic outputs.

Here, we provide the rationale for and details of the considered techniques, along with an account of the design choices governing our model's development. We present an evaluation of the resulting trained model using real-world field recordings. This study's contributions extend beyond the confines of katydid recognition. It highlights the importance of adopting tailored approaches in developing machine-listening solutions, with greater emphasis on data engineering to surmount common data-related challenges. The proposed techniques are generic and parametric, and provide a robust and adaptable approach that caters to researchers looking to develop insect biodiversity monitoring solutions.

## 2. Material and methods

### (a) Training dataset

At the start of our study, we had access only to a limited set of focal recordings from a prior study whose goal was to describe call characteristics of katydids on BCI [33]. The focal recordings were obtained from caged male katydids placed in a controlled quasi-natural setting resembling their natural environment. Acoustic foam was used to suppress sound reflections and limit ambient noise. Audio recordings were collected at a sampling frequency of 250 kHz using a CM16 condenser microphone (AviSoft Bioacoustics, Germany) placed at 30 cm from the focal insect and connected to an UltraSoundGate 416H A/D converter (AviSoft Bioacoustics, Germany). The resulting data presented high-fidelity recordings of katydid sounds with minimal background noise and other interferences. With little being known of the call characteristics of Panamanian katydids prior to ter Hofstede *et al*. [33], their protocol for focal data collection ensured utmost confidence in establishing call-to-species associations. However, the ‘domain mismatch’ of these near-studio-quality recordings with typical field recordings rendered them far from ideal as a standalone training dataset for training ML models. Furthermore, the focal recordings contained very few instances of any discernible non-katydid sounds, the lack of which limits an ML model's ability to learn to reject ‘out of vocabulary’ sounds.

To overcome the above shortcomings, we augmented the training dataset with *in* *situ* ambient recordings. Using Swift autonomous recorders (K. Lisa Yang Center for Conservation Bioacoustics, Cornell University) programmed to record at a sampling rate of 96 kHz, we recorded BCI soundscapes at different times of day on 24 different days over a span of seven months in 2019. Following manual annotations (see below), we retained approximately 1.3 h of recordings that contained katydid sounds and other discernible sounds from the environment. Although these recordings would enable the ML model to better adapt to the target application environment for which it is being developed, they however contained calls of only 22 of the 31 target species. Also, for many species, the numbers of detected calls were low (13 species with fewer than 10 calls; 10 species with fewer than 3 calls).

To address the above limitations, we further augmented the training set using playback recordings, wherein katydid sounds from the focal recordings were played back using an Avisoft Bioacoustics Vifa speaker with an Avisoft USGH 216 amplifier and re-recorded using a Swift recorder. Prior to playback, the speaker was characterized using a tone sweep, and playback recordings were filtered to compensate for deficiencies in speaker response. To mimic differences in attenuation and variations in spectrographic features arising from the propagation of sound in vegetated environments, the transmitter and the recorder were placed in six different spatial configurations among bushes and leaf cover and at varying relative distances (in the range 1–6 m).

Following an annotation protocol similar to that of Symes *et al*. [50], we produced call-level annotations using Raven Pro v.1.6 [51] for all three recording sets (i.e. focal recordings, *in situ* recordings and playback recordings). Annotations comprised boxes bounding the calls' time (within respective files) and frequency extents. In the *in situ* recordings, we also annotated calls of bats (one of the dominant predators of katydids) and other non-katydid sounds commonly occurring in the environment. With bats’ echolocation clicks having some resemblance to pulsed calls of a few katydid species (e.g. *Anaulacomera furcata*, *Anaulacomera* ‘ricotta’, *Anaulacomera* ‘goat’, and *Anaulacomera spatulata*) and with both occurring in similar bandwidths, having a separate detection-target class for bats facilitates training the ML model to better discriminate these and thereby helps us easily discard bat calls from detection outputs. Similarly, annotations of other non-katydid sounds facilitate having a ‘catch all’ reject class. Further, we also annotated representative time periods that did not contain any discernible spectrographic components. Besides facilitating training the ML model not to produce high scores in the absence of target sounds, these recording periods were also used during data augmentation (§2c(ii)).

### (b) Test dataset

Our test dataset comprised field recordings sampled pseudo-randomly from a long-term katydid monitoring programme at BCI. Audio recordings were collected at two locations on BCI using Swift recorders placed at a height of 24 m in the forest canopy. Vegetation density varied notably between the chosen locations, offering the potential to capture a wider range of possible species variations. The recorders were programmed to record for ten minutes at the beginning of each hour from dusk until dawn, at a sampling frequency of 96 kHz. From a period spanning five months (in 2019) covering both wet and dry seasons, we selected five dates corresponding to new moon nights. Katydids are known to be most active during darker nights [52,53]. From the 5 days, we randomly selected three 10-minute recordings representative of soundscapes immediately after dusk, at midnight and shortly before dawn.

Using Raven Pro software, we manually annotated the selected recordings using a dual-observer dual-reviewer protocol [50] wherein each audio file was initially annotated by one of the two observer analysts and was then reviewed iteratively by two expert analysts. Data corruption due to equipment malfunction at one of the sites during a dry season day forced us to discard three audio files from the corresponding day. The final test dataset contained a total of 270 min of soundscape recordings with annotations for 24 species of katydids, out of which 21 were common with our list of target species.

### (c) Data engineering

The calls of *Anaulacomera* ‘wallace’ and *Hetaira sp.* exhibit extreme similarity and are indistinguishable to human analysts. For the purposes of automatic classification, we merged annotations from these two species into a single class. We also combined annotations of all the uniquely identified non-katydid sounds into a single ‘catch all’ reject class. In total, we had 32 detection-target classes, of which 30 corresponded to 31 katydid species, one to bats, and one to the ‘catch all’ reject class. Annotations of the ‘background’ category were not assigned to a detection-target class. Instead, for training inputs (§2c(i)) belonging to this category, we set their ground-truth labels (a 32-dimensional vector containing values in the range 0–1) to contain all zeros.

#### (i) Input preparation

Considering that call energies of all the katydid species considered were dominantly above approximately 7 kHz, we applied a 12th order Butterworth high-pass filter to suppress energies below 6.9 kHz. For consistency and speed-up of downstream steps, we downsampled all audio recordings to a sampling frequency of 96 kHz. This resulting Nyquist rate (48 kHz) remained well above the dominant frequencies of the calls of all included katydid species. Furthermore, this choice simplifies the envisaged application of the trained model resulting from this study—field recordings would be gathered at 96 kHz sampling frequency and as such no resampling would be necessary then. For producing fixed-dimension inputs to the CNN model, first we split up the downsampled continuous audio recordings into segments of 0.8 s duration. By considering different amounts of segment advance (= segment length – segment overlap), we coarsely controlled the number of segments generated in different scenarios. When pre-processing focal recordings, we considered different segment advance amounts for different species groups (table 1). These choices were driven primarily by the number of available annotations for each species and were aimed at reducing the magnitude of class imbalances in training inputs. For pre-processing *in situ* and playback recordings, we considered a fixed segment advance amount of 0.2 s. This pre-processing of the *in situ* recordings also generated ‘background’ segments, corresponding to annotations from that category. Additionally, we repeated pre-processing of *in situ* recordings with a segment advance of 0.1 s and only retained segments corresponding to ‘background’ annotations. These are ‘additive background’ segments for use in data augmentation (§2c(ii)) and do not constitute class-assigned training inputs.

**Table 1.** Parameters considered in pre-processing of focal recordings corresponding to different species. Chosen segment advance amounts included 0.05 s (♣), 0.15 s (♦), 0.2 s (♥), 0.4 s (♠), 0.55 s (□) and 0.6 s (▪). Sound centralization penalty was considered for 13 of the 31 species.

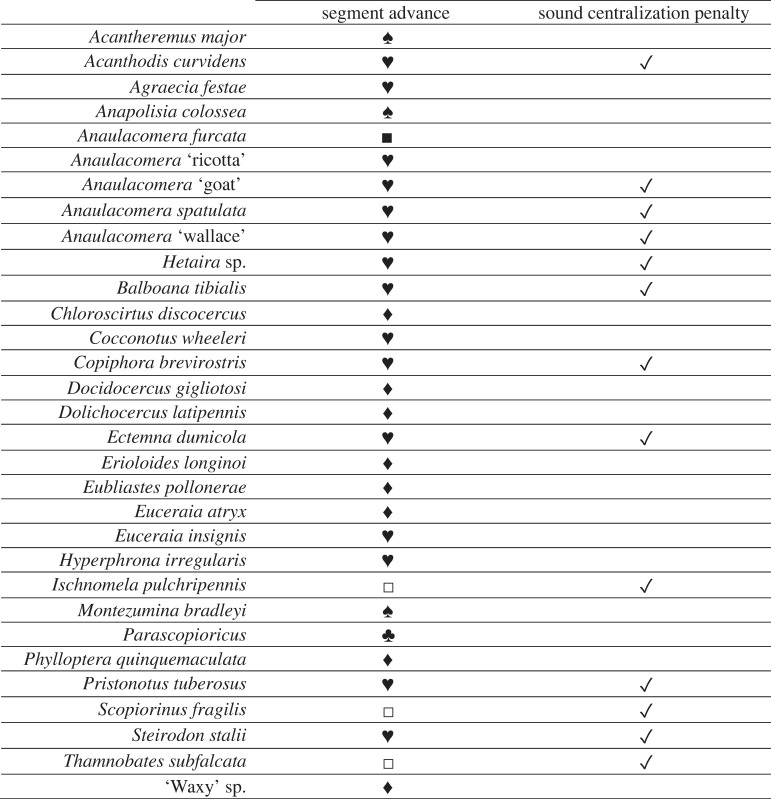

We set the per-class ground-truth scores (values in the range 0–1) in the 32-dimensional label vectors for each segment to be reflective of the amount of temporal overlap between the segment and annotations from the corresponding class—where the segment fully contained an annotation or *vice versa*, we set the ground-truth score to 1.0, and where there was partial overlap between the segment and an annotation, we divided the overlap duration by the shorter of 0.8 s or the annotation's duration. For certain species with very short calls (annotations shorter than 50% of segment duration, i.e. 0.4 s), we applied an additional ‘sound centralization’ penalty—for annotations that neither occurred within the central 25–75% of the segment nor spanned across the mid-epoch of the segment, we penalized the ground-truth score by a factor of (1 − *δ*/0.4), where *δ* is the shortest temporal distance from the segment centre to the annotation. We applied sound centralization penalty only to segments from focal recordings.

Power spectral density spectrograms of normalized audio segments (waveform values in the range [−1.0, 1.0]) were computed using a 5 ms Hann window with 50% overlap between frames, resulting in time- and frequency resolutions of 2.5 ms and 200 Hz, respectively. We clipped the spectrograms along the frequency axis to retain only portions between 6.8 kHz and 47.4 kHz, which resulted in model inputs having dimensions of 204 × 319 (*height* × *width*).

The generated audio segments and the associated ground-truth scores (labels) constituted intermediate outputs of the input preparation stage. Although conceptually part of the input preparation stage of typical bioacoustic ML workflows, computations to transform audio segments into spectrograms were performed on-the-fly as a part of the training process (§2d(ii)) to facilitate ease in applying different types of data augmentations.

#### (ii) Data augmentation

Given our modest-sized training dataset, we employed a variety of parametric data augmentation techniques (described below) to meaningfully inflate the training set while also improving the variance among the training inputs. These included both time-domain and spectral-domain augmentations. We set the sequence and probability of application of each augmentation type differently for focal and non-focal (*in situ* and playback) training inputs (table 2). We also selected different ranges of values for the parameters of each augmentation type for different input types. We made these choices (i.e. sequence and probability of application, parameter ranges) empirically, informed by our knowledge of the considered calls and while ensuring that the augmentations remained label-preserving. In the below descriptions of the considered augmentation types, all ‘random choices’ arise from a uniform distribution within the respective ranges.

**Table 2.** Probability of application of time-domain^a^ and spectral-domain^b^ augmentations to training inputs from focal and non-focal (*in situ* and playback) recordings. The ordering in the table reflects the sequence of application of the augmentations. ^c^For focal inputs, Gaussian noise was only added to 25% of the inputs among those 33% that were not subject to background infusion. [NA: Not applied.]

augmentation	probability of application	
	focal	non-focal
*echo*^a^	0.20	NA
*volume ramping*^a^	0.33	0.33
*background infuse*^a^	0.67	NA
*Gaussian noise*^a^	0.25^c^	0.75
*variable noise floor*^b^	1.00	1.00
*alter distance*^b^	0.33	0.25

*Echo*. Synthesizes echo effect by adding a dampened and delayed copy of the input signal's waveform to itself. The dampening factor—a scalar multiplier applied across the copy—reflects a desired attenuation of *l* dB. The dampened copy is added to the original waveform with an offset of *t* ms. We set *l* to vary randomly in the range [−24, −15] and *t* to vary randomly in the range [1, 6].

*Volume ramping.* Simulates the effect of a source moving away from or closer to the receiver, by multiplying the input segment's waveform with an amplitude envelope that linearly decreases or increases, respectively. Setting one end of the amplitude envelope to 1.0, it is linearly reduced to a lower value (less than 1.0) at the other end to reflect an attenuation of *l* dB at that end. We set the ramping direction to vary randomly (by randomly selecting the end to be attenuated) and set *l* to vary randomly in the range [0, 6].

*Background infuse.* Adds real-world ambient noise to training inputs from focal recordings. The waveform of a randomly selected segment from the ‘additive background’ set is added to that of a focal segment after subjecting the former to *volume ramping* augmentation followed by attenuation. The attenuation, wherein a scalar multiplier is applied across the noise segment to reflect an attenuation level of *l* dB relative to the absolute peak in the focal segment, ensures that target signals in focal segments retain dominance in the resulting outputs. We set the ramping parameter (see above) to vary randomly in the range [0, 9] and set the attenuation level *l* to vary randomly in the range [−18, −12].

*Gaussian noise.* Adds Gaussian noise to the input segment's waveform. We set the standard deviation of the distribution such that the resulting noise waveform's maximum absolute amplitude occurred at a level that was *l* dB below the absolute peak amplitude in the signal waveform. We set *l* to vary randomly in the range [−24, −15] for segments from focal recordings and in the range [−45, −30] for segments from non-focal recordings.

*Variable noise floor.* Mimics the effect of the foreground having different loudness levels relative to the background. Typically, before converting linear-scale spectrograms to log-scale, a small positive quantity *ε* is added to avoid computing log(0). Our choice of *ε* = 1 × 10^−10^ produces spectrograms with a noise floor of −100 dB full scale. During training, we set *ε* to vary randomly to reflect a noise floor in the range [−105, −85] dB full scale.

*Alter distance.* Mimics the effect of increasing or decreasing the distance between a source and the receiver by attenuating or amplifying, respectively, a spectrogram's components at higher frequencies. A quantity whose magnitude varies linearly from 0 to *l* dB over the bandwidth of the clipped spectrogram (see §2c(i)) is added to all frames of the spectrogram, with the values 0 and *l* dB corresponding to the lowest and highest frequencies, respectively. We set *l* to vary randomly in the range [−6, −3] for segments from focal recordings and in the range [−3, 3] for segments from non-focal recordings. For non-focal recordings, the range spanning both negative and positive quantities simulated both increases and decreases in source-to-receiver separation. Attenuation (only) factors were set higher for focal recordings given the known smaller separations.

### (d) Machine learning

We built, trained and tested our ML model using Koogu (v.0.6.2; https://pypi.org/project/koogu/0.6.2/), an open-source framework for machine learning in bioacoustics. The underlying deep-learning framework was TensorFlow (v.2.3.1; [54]), running on Python v.3.6 (Python Foundation). We performed training and testing on an Ubuntu 20.04-based computer featuring an octa-core Intel Xeon Silver 4110 processor, 96 GB RAM and an NVIDIA Tesla GPU with 16 GB video RAM.

#### (i) CNN architecture

We chose a quasi-DenseNet architecture [55] due to its computational efficiency and ability to train well with few samples. With 3 × 3 pooling at transition blocks (between successive quasi-dense blocks), the spatial dimensions of model inputs (204 × 319) diminish down to 3 × 4 over five blocks, rendering the resulting shape small enough to apply global average pooling. As such, we implemented a CNN with five quasi-dense blocks, each having two layers per block with a growth rate of 32. We added a pre-convolution layer having 32 3 × 3 filters preceding the five-block network. We connected the outputs of the five-block network to a global pooling layer, which was followed by a 64-node fully connected layer, a batch normalization layer [56], a ReLu activation layer [57] and finally, a 32-node (corresponding to 32 classes) fully connected layer with sigmoid activations.

#### (ii) Training

We used 90% of the prepared segments for training the model and the remaining 10% for evaluating through the training process. We weighted training losses appropriately to address class imbalance in the training inputs. To improve model generalization, we used dropout [58] with a rate of 0.05. We trained models over 60 epochs using the Adam optimizer [59] and with a mini-batch size of 32. We used an initial learning rate of 0.01 and successively reduced it by a factor of 10 at epochs 20, 40 and 50.

To assess the level of influence that the considered augmentation techniques and the added playback recordings had on recognition performance, we considered training additional models (i) without the parametric augmentations and playback set (considering only the original focal and *in situ* recordings), and (ii) with parametric augmentations but without the playback set. To ascertain consistency, we replicated training in the three scenarios (i.e. only original recordings, original recordings + augmentations, and original recordings + playback recordings + augmentations) five times, producing a total of 3 × 5 = 15 fully trained models. For some level of completeness, we also included a transfer learning experiment using a MobileNetV2 [60] model whose weights were pre-trained on the ImageNet dataset [61]. Single-channel spectrograms were transformed into 3-channel equivalents using the jet colour palette, and were resized to 224 × 224 before being fed to the model. Limiting the transfer learning experiments to only the ‘all in’ scenario (i.e. original recordings + playback recordings + augmentations), we also trained five replicates of the transfer learning model. To ensure that all four models (three quasi-DenseNet + one MobileNetV2) in a replication set trained from the same ‘starting point’, we deterministically seeded the randomized initialization of neural-network weights (seed values were same for the different training scenarios in each replication, but differed across the five replications).

### (e) Inferencing protocol

We subjected recordings from the test dataset to the same sequence of pre-processing operations as the training dataset (i.e. resampling, filtering, segmentation, label association and spectrogram generation), but did not apply any augmentations. In contrast to the ad hoc amounts of segment advance chosen when generating training inputs, we used a fixed segment advance of 0.2 s to generate test inputs.

**Figure 2.**
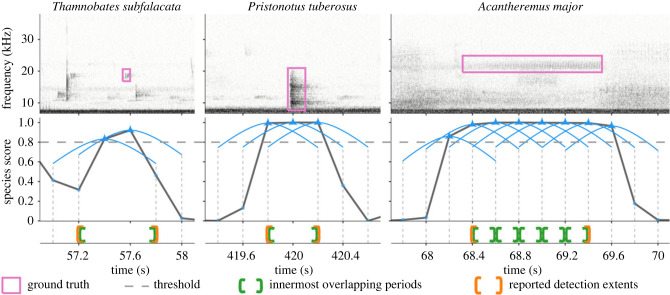
Illustration of the post-processing algorithm using actual test data (top row) and model outputs. The middle row shows raw (as generated by the model) per-segment scores (for the corresponding species), with blue triangles (temporally centred within the segments’ time extents) indicating scores exceeding the detection threshold (0.8; chosen for demonstration). Blue arcs extending on either sides of the triangles, along with the dotted vertical lines dropping down from their tips, show the time extents of each segment. The bottom panel shows, for each example (columns from left to right), the different numbers of innermost overlapping periods present (1, 1 and 5, respectively, resulting from 2, 3 and 8 successive segments exceeding the threshold) and the time extents of the combined detections reported by the algorithm.

Given the wide range of durations of the target calls (from a few milliseconds to multiple seconds; electronic supplementary material, figure S1) and their disparity with the chosen segment length, we employed a post-processing algorithm to convert raw segment-level detections (with segment boundaries as a detection's time extents) into ‘grouped and processed detections' whose temporal extents were better aligned with those of the detected targets (figure 2). Applying a detection threshold *τ* to the model's outputs, for each class we identified groups of contiguous segments having detection scores ≥*τ*. Given the chosen values for segment length and advance, the innermost overlapping periods within a group were 0.2 s, 0.4 s or 0.6 s long for groups of 4, 3 or 2 segments, respectively, with 4-segment groups having the most-overlapped periods. Treating each group as a single detection, the post-processing algorithm reported time extents of the innermost overlapping periods as the detection's time extents. This logic catered to short-duration targets (less than 0.8 s, such as the calls of *Anaulacomera* ‘goat’, *Anaulacomera furcata*, etc.; see left and centre columns in figure 2). A larger grouping (more than 4 segments) resulted in a contiguous sequence of 0.2 s-long most-overlapped periods. Treating each larger grouping as a single detection, the post-processing algorithm reported the period encompassing the first and last of the corresponding most-overlapped periods as the detection's time extents. This logic catered to longer-duration targets (greater than 0.8 s, such as the calls of *Acantheremus major*, *Erioloides longinoi*, etc.; see right-most column in figure 2). Treating positive segments (segments having scores ≥*τ*) that were not part of a grouping as independent detections, the post-processing algorithm reported the segments' temporal boundaries as detection time extents. We retained all reported detections for later analysis.

### (f) Performance assessment

Given our inference protocol, time extents of reported detections were always multiples of 0.2 s. However, annotations of ground-truthed (GT) calls had no such constraints, which made automated matching of detections to annotations a non-trivial endeavour. Attempts to automate were further complicated (i) by the large variations in the durations of both detections and annotations, and (ii) for closely spaced short-duration GT calls, the post-processing algorithm could report fewer (or just one) detections each encompassing multiple GT calls. We therefore employed certain ‘coverage’ constraints to classify each reported detection as either a true- or a false-positive (TP or FP, respectively) and to ascertain whether a GT call was recalled or missed. For each reported detection, if it had any temporal overlap with one or more GT annotations of the same class and if the sum of the overlapping durations exceeded 60% of the detection's duration, then the detection was considered a TP. Similarly, a GT call was considered ‘recalled’ if its annotation had any temporal overlap with one or more reported detections of the same class and if the sum of the overlapping durations was ≥50% of the annotation's duration. Note that these rules for matching led to a deviation from the popular way of determining a detector's rate of recall—owing to an absence of direct correspondence between TPs and GT annotations (therefore TPs + false negatives ≠ GT calls), we simply take the ratio of the number of recalled GT calls to the total number of GT calls. To assess the precision versus recall characteristics of the trained model, we set *τ* to values in the range 0–1 (intervals of 0.05), and for each threshold value, we applied the post-processing algorithm and computed precision (TP/(TP+FP)) and recall rates.

### (g) Manual validation

We reviewed reported detections that were not matched to any GT annotations. In a substantial number of cases, these detections corresponded to faint calls that the analysts had either missed or did not annotate because they did not fit the ‘visible pulse structure’ criterion [50]. Often, the calls were sufficiently faint that they were not readily visible in spectrograms. By filtering the recordings using a bandpass filtre (with appropriate pass-bands for different species) and adjusting visualization settings (in Raven Pro) appropriately, we were able to verify the signatures of many of the reported detections in spectrogram or in waveform, or both (for an example, electronic supplementary material, figure S2). In other cases, the reported detections corresponded clearly to notable call-like spectrographic features, but with call structures that were sufficiently degraded that human observers could neither support nor refute the model-assigned classes. Finally, there were cases where no signal was observable despite intensive review (absolute false positives).

## 3. Results

**Figure 3.**
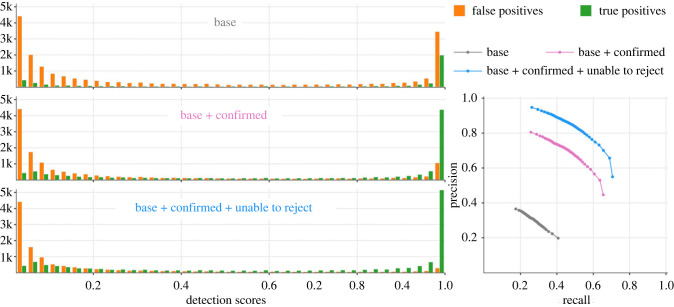
Recognition performance of the trained model against the test dataset—distribution of model's score outputs (left) categorized into and true- and false-positive detections, and precision-recall characteristics (right). In practice, the actual performance of the model lies between the ‘base + confirmed’ and ‘base + confirmed + unable to reject’ assessments.

Applying the different segment advance choices to the training dataset generated 10 989 segments that were assigned to one or more species classes (see electronic supplementary material, table SI for a breakdown of per-class counts), 1057 ‘background’ segments and 2101 ‘additive background’ segments. With 10% of these carved out for validation, there were less than 10 000 species-assigned training samples. Having only 476 k total and 471 k trainable parameters, our 15-layer quasi-DenseNet model was considerably smaller compared to popular off-the-shelf architectures such as MobileNetV2 (53 layers, >2 million parameters) and EfficientNetB0 (>4 million parameters; [62]). Training achieved convergence for all 15 + 5 models. The quasi-DenseNet models attained final training and evaluation accuracies of up to 99.68% and 99.64%, respectively, with respective binary cross entropy losses as low as 4 × 10^−3^ and 8.9 ×10^−3^ (figure 3 and electronic supplementary material, table SII). The fine-tuned MobileNetV2 models attained final training and evaluation accuracies of up to 98.81% and 98.46%, respectively, with respective binary cross entropy losses as low as 2.5 × 10^−2^ and 4.9 × 10^−2^ (electronic supplementary material, figure S6 and table SIII).

As the primary goal of the study was to develop a well-performing trained model for analysing field recordings, we limited the painstaking manual validation efforts (§2g) to just one of the models from the ‘all in’ scenario. Following manual analyses of detections generated by the model on test set recordings, we report three versions of performance assessments. The first version, labelled ‘base’, matched reported detections to original annotations of the test recordings. In the second version, labelled ‘base + confirmed’, we added to the TP set (and to the GT set) any apparent FP detections that human observers could confidently match to underlying call(s), and present model performance using the expanded GT set. For the third version, we also treated as TPs any detections that could not be confidently rejected as FPs. In practice, the true performance of the model lies between the latter two assessments. While the model identified with high scores a large number of faint and degraded calls that human analysts had originally overlooked, the majority of the wrong classifications remained concentrated at low scores (figure 3). These are good indicators of the model's robustness in processing real-world field recordings.

**Figure 4.**
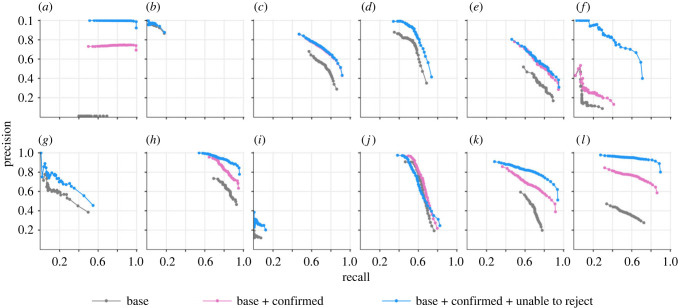
Class-specific precision-recall curves for katydid species that had at least 20 ground-truth annotations in the test dataset—(*a*) *Acantheremus major*, (*b*) *Agraecia festae*, (*c*) *Anaulacomera furcata*, (*d*) *Anaulacomera spatulata*, (*e*) *Anaulacomera* ‘wallace’ or *Hetaira sp*, (*f*) *Docidocercus gigliotosi*, (*g*) *Ectemna dumicola*, (*h*) *Erioloides longinoi*, (*i*) *Ischnomela pulchripennis*, (*j*) *Montezumina bradleyi*, (*k*) *Pristonotus tuberosus*, and (*l*) *Thamnobates subfalcata*. The apparent absence of pink lines in some of the panels is due to obscuration by blue lines as there were no detections of the corresponding class that the human observers could neither confirm nor refute.

A class-specific breakdown of the recognition performance (figure 4) shows the discriminative power of the model in distinguishing between closely related call types (e.g. among congeneric Phaneropterinae *Anaulacomera* ‘wallace’ and *Anaulacomera furcata*). The model recognized with high accuracy the longer calls of Conocephalines (e.g. *Acantheremus major*, *Erioloides longinoi*) and loud calls of certain Pseudophyllinae (*Pristonotus tuberosus* and *Thamnobates subfalcata*). The lower overall recall rate of the model (figure 3) may be attributed to low recall rates for species having a large number of annotations in the test set, such as *Agraecia festae* (1155) and *Ischnomela pulchripennis* (1622). Among the species having no original annotations in the test dataset, no TP detections were reported for *Balboana tibialis*, *Copiphora brevirostris* and *Steirodon stalli* whereas for *Paracopioricus* and *Eubliastes pollonerae* the model attained moderate-to-high F1-scores (electronic supplementary material, figure S4).

**Figure 5.**
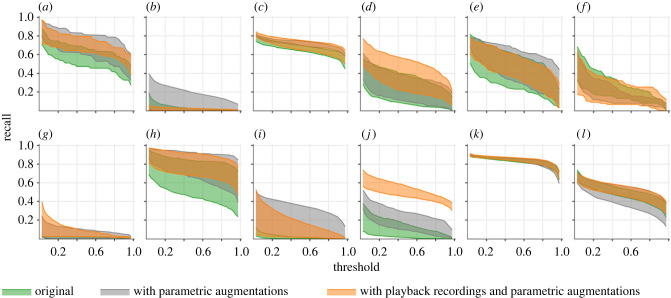
Class-specific recall rates (panels *a*–*l* correspond to the respective classes listed in figure 4). Upper and lower extents of the filled regions correspond to maximum and minimum recall rates, respectively, obtained for the five quasi-DenseNet models in each training scenario.

The manual validation exercise (§2g), which categorized each reported detection (that did not have a match with GT annotations) as either TP or FP, had a larger influence on the model's precision rates than it had on recall rates as the categorization affected both the numerator and the denominator in the ratio TP/(TP+FP). Given our approach to computing recall rates (see §2f), we could still obtain a representative estimate of a model's recall in the absence of manual validation. As such, we present performance comparisons between quasi-DenseNet models from the three training scenarios (§2d(ii)) using only their esimated recalls (figure 5). For better clarity with making pairwise comparisons, see electronic supplementary material, figure S5. Comparisons against the transfer learning approach, which was not among the primary objectives of the study, are also included in electronic supplementary material (figure S7). Given that we did not conduct any formal search for optimal hypermater combinations, readers are advised to treat these comparisons as indicative.

## 4. Discussion

In this study, we addressed the non-trivial task of developing a ML detector for neotropical katydid sounds. Our efforts highlighted two overarching categories of challenges: domain-specific challenges and data-related constraints. These are not unique to our work but are emblematic of the broader challenges faced when developing machine listening solutions for multi-species recognition within the domain of insects.

The first category of challenges stems from the unique characteristics of katydid calls. In contrast to avian or marine mammal recognition, where established solutions exist, katydid recognition in tropical soundscapes presents a distinctive set of complexities. Given the extreme variance in the temporal, spectral and spectro-temporal characteristics of the calls of neotropical katydids, automatic recognition of certain species' calls is akin to ‘finding a needle in the haystack’. For example, the pulse durations of Phaneropterinae (0.8–1.7 ms) are often far narrower than typical frame widths considered in computing spectrograms. On the other hand, inter-species discriminatory traits sometimes diminish to subtle differences in factors such as pulse repetition rates (e.g. *Euceraia atryx* versus *Euceraia insignis*) and peak frequencies (e.g. *Anaulacomera furcata* versus *Anaulacomera* ‘ricotta’; see figure 1 for examples). Such extreme conditions precluded the use of pre-trained models (more on this below) or off-the-shelf solutions like BirdNET (with its 3-second-long, limited bandwidth inputs [63]). The idiosyncrasies of tropical insect calls necessitate an approach that comprises meticulously tailored processing at all facets of an ML workflow.

The second category of challenges emerged from the nature of the available training dataset—relatively small size, imbalanced class distribution and atypical origins—which presented limited resources for ML model development. While some classes had very few annotated calls (e.g. *Erioloides longinoi* and *Parascopioricus*), many classes contained only sounds from just one (e.g. *Acantheremus major*) or a few individuals, presenting significant class representativeness issues. Furthermore, the primary recordings in the dataset were collected in controlled environments that minimized ambient noise, deviating from the real-world soundscapes where the model would ultimately be deployed.

Use of pre-trained computer vision (CV) models in bioacoustics often neccesitates resizing of spectrograms to match a model's input shape expectations. Commonly available pre-trained models typically support inputs of shape 224 × 224 and 96 × 96. While upsizing spectrograms may have little or no adverse effects, downsizing could introduce artefacts and the ensuing compression along one or both axes could lead to distortion of discriminatory traits. Few other studies have explicitly considered the impacts of input compression (e.g. [64]). In our case, we chose the option of 224 × 224 as it was the closest match and would result in the least compression—approximately 30% along the horizontal (time) axis and none along the vertical (frequency) axis. Such a high compression ratio (1 : 0.7) across the time axis would cause, among other performance-lowering effects, a collapse of the closely spaced multi-pulse structure of some Phaneropterinae calls (especially *Anaulacomera* ‘wallace’/*Heitara sp*.) and a squishing of the spectral features in the short-duration frequency-modulated calls (see [33]) of *Copiphora brevirostris*, *Docidocercus gigliotosi*, *Eubliastes pollonerae*, *Montezumina bradlyei* and *Thamnobates subfalcata*, resulting in a loss of their characteristic distinctiveness. Deviating from the common practice of adopting very large CV models in bioacoustics, a few other studies have also demonstrated the upshot of using bespoke fully trained CNN architectures (e.g. [55,65,66]). Our choice of using a modest-sized CNN not only made training and inferencing fast, but it also reduced any risk of potential overfitting given the small training dataset. While adding to the small, but growing, body of literature on using custom-designed CNNs in bioacoustic pattern recognition, this study also highlights the importance of increased emphasis on data engineering (preprocessing, class-balancing, input conditioning, near-realistic augmentations, etc.) in non-trivial recognition problems.

Our choice of a 0.8 s segment length for breaking up continuous audio was a judicious selection as it strikes a balance between competing considerations. It is just long enough to capture sufficient portions of longer-duration calls (>0.8 s; e.g. *Acantheremus major*, *Erioloides longinoi*) to be able to unambiguously identify the source. On the other hand, it is short enough for the shortest of calls (e.g. Phaneropterinae calls) to manifest as significant portions of model inputs. For species with variable number of pulses (e.g. *Anapolisia colossea*, *Thamnobates subfalcata*), the 0.8 s window ensured that each input captured enough (≥4) pulses of the full call. This was vital, especially where inter-pulse intervals were a strong discriminatory trait (e.g. *Euceraia atryx* versus *Euceraia insignis*). While a 0.2 s segment advance during inferencing ensured that calls present in the recordings were captured by one or more segments, the *ad libitum* segment advance settings considered during input preparation improved class balance among training inputs. The choice of 0.8 s segments was also a trade-off between larger inputs, which exact higher resource demands and slower processing, and smaller input segments, which may not capture sufficient discriminatory traits. When associating ground-truth values to training inputs, our choice to assign continuous (non-binary) values ensured that inputs with clipped calls (at segment boundaries) were penalized less during training. Consequently, the models also likely learned better to produce partial scores for inference inputs with truncated calls. The latter benefit was also conducive for the post-processing stage.

Many studies have considered audio conditioning and spectrogram generation as part of the ‘input preparation’ process (e.g. [4,67]), generating spectrograms as intermediate outputs (often stored as image files). A few studies have considered applying augmentations also as part of the same process (e.g. [68,69]), generating augmented replicates of the original training set. Such approaches, besides increasing storage needs, make applying dynamic time-domain augmentations problematic. Breaking up the input preparation workflow into disjoint processes—audio pre-processing (resampling, filtering, segmentation and label-association) and on-the-fly spectrogram generation—allows additional flexibility in applying both time-domain and spectral-domain augmentations (e.g. [70]). By saving pre-processed audio segments (instead of spectrograms) as intermediate outputs and deferring the audio-to-spectrogram transformation to form part of the data-flow pipeline during training, we could easily include computations for a number of on-the-fly temporal and spectral augmentations prior to and post spectrogram generation, respectively. By randomizing the probability-of-application of each of the chained augmentations and by randomizing the parameter values of each augmentation type, we were able to greatly improve variance in the training inputs, both within each training epoch as well as across epochs.

Given the subtleties in the discernible traits of katydid sounds, synthetic masking (even minimal) risks occluding significant portions (sometimes, the entirety) of short-duration or short-bandwidth calls, thereby precluding augmentation techniques such as SpecAugment [71]. The short duration of input segments left little scope for applying time-warping augmentations without considerably risking label alteration or annulment. Also avoiding commonly used techniques from the CV domain (like rotation, reflection) that could result in label-altering outcomes, we considered only simplistic and largely physics-based augmentation techniques whose outputs resembled naturally occurring conditions more closely and were therefore more label-preserving. The considered data augmentations resulted in non-negative improvements in recall for every class (having more than 20 ground truth annotations in the test set), with the exception of *Docidocercus gigliotosi*. The positive improvements were noteworthy in the case of *Agraecia festae*, which had training inputs only from the focal set, and *Ectemna dumicola*, which had no training inputs from the *in situ* set and only six from the playback set (see electronic supplementary material, table SI). Though *Pristonotus tuberosus* had the least number of training inputs (64) in the focal set, it had attained high recall rates even without augmentations, as the high number of instances in the *in situ* set (219; the highest for any species) are likely to have provided adequate variance and coverage of target domain's characteristics. Other classes having high numbers of instances in the *in situ* set also saw little (*Anaulacomera furcata*) to no (*Thamnobates subfalcata*) improvements from augmentations, with the exception of *Acantheremus major*, which had a notable bump in recall. In the case of *Montezumina bradleyi*, which had no instances in the *in situ* set although its recall improved notably due to data augmentation, the improvements were dwarfed considerably by the inclusion of training inputs from the playback set. In the case of *Acantheremus major* and *Erioloides longinoi* (both having long-duration calls), with recall already attaining quite high values following augmentations there was little scope for the inclusion of playback set to improve performance any further. The very short-duration calls of Phaneropterinae species *Anaulacomera furcata* and *Anaulacomera ‘*wallace’ may likely have suffered intense signal degradation during the playback-recapture exercise (either from propagation and/or when the original recordings were filtered). Small dips observed in their recall rates may be attributable to this phenomenon. Further, we observed a strange behaviour where recall rates for some classes that had no training inputs in the playback set changed notably when models were trained with the playback set—recall rates of *Agraecia festae* and *Ischnomela pulchripennis* decreased, while that of *Anaulacomera spatulata* increased. These warrant further investigation. While we observed some improvements from the inclusion of playback set, its influence across classes was not as consistent as that of data augmentations. In addition to potential signal degradation issues (see above), our playback set was also not well-rounded, providing coverage for only half of the classes and having very few samples for some classes. A meaningful expansion of the playback set with additional recaptured recordings may help stabilize performance improvements. Alternatively, one could also simulate a playback set by convolving the focal recordings with impulse responses representative of the target environments that one attempts to mimic [72]. Such a technique offers a cost-effective alternative to using physical transmitters and receivers and offers the ability to ‘add’ newer environments to a playback set without the need for physical access to those environments.

As for the additional experiments with pre-trained models, training (and evaluation) accuracies and losses came close to those of the corresponding fully trained quasi-DenseNet models. However, recall rates were significantly lower (*p*-value < 10^−3^) across all thresholds for three classes (*Anaulacomera furcata*, *Montezumina bradleyi* and *Thamnobates subfalcata*) and considerably lower for most other classes. These findings provide credence to our concerns regarding the impacts of reducing spectrogram dimensions to suit an available model.

Starting with a dataset that was dominated by ‘studio quality’ focal recordings (see figure 1 for examples), we have successfully developed an ML solution capable of analysing real-world field recordings. The model's performance underscored the effectiveness of our data engineering efforts. While it excelled in the recognition of species with longer calls (high precision and recall), certain species, particularly those with pulsed and repetitive calls, posed challenges to its rate of recall. This observation may, in part, reflect post-processing considerations and warrant further investigation. We acknowledge that, despite offering good coverage of environmental conditions, the considered test set proved less substantial with respect to species coverage—it contained few or no calls from some of the target species. Subsequent work could consider expanding the test set with additional annotated field recordings to improve significance in assessments. However, we performed additional empirical checks on several random recordings from the area and noted promising outcomes—few FPs and few missed calls, and high confidence (scores close to 1.0) for most TPs. Further, in a related study [73], large-scale analyses of multi-year recordings were performed using the trained model from this study, and daily calling patterns (inferred based on reported detections) of several species were found to be in close agreement with prior knowledge.

Our work not only addresses the specific challenges of katydid sound recognition but, by successfully navigating the idiosyncrasies of domain-specific signal processing and data-related constraints, it also offers a valuable foundation for future endeavours in the realm of insect biodiversity monitoring through machine listening. The design considerations and techniques developed in this study, being generic and parametric, can be readily adopted and adapted by other researchers and initiatives focused on developing machine listening solutions for insect biodiversity monitoring. In our attempt to enrich the resource pool available to the broader scientific community, we have integrated the developed techniques into recent releases of the open-source Koogu toolbox and have also bundled the final trained model with releases of Raven Pro, starting from v.1.6.5.

## Data Availability

Data considered in this study were from previously published works [33,50]. The techniques developed in this study have been integrated into recent releases of the open-source Koogu toolbox: https://github.com/shyamblast/Koogu. The final trained model is also bundled with releases (starting from v.1.6.5) of Raven Pro software. For replication of the results presented in the manuscript, all training audio (focal, *in situ* and playback) and annotations (in Raven Pro selection table format) can be obtained from the Zenodo Repository: https://doi.org/10.5281/zenodo.10594015 [74] and all testing audio files and annotations (also Raven Pro format) are available from the Dryad Digital Repository: https://doi.org/10.5061/dryad.zw3r2288b [75]. Additional data and results are provided in electronic supplementary material [76].
